# Training modulates memory-driven capture

**DOI:** 10.3758/s13414-022-02508-0

**Published:** 2022-06-09

**Authors:** Edyta Sasin, Florian Sense, Mark Nieuwenstein, Daryl Fougnie

**Affiliations:** 1 Department of Psychology, New York University of Abu Dhabi, Abu Dhabi, United Arab Emirates; 2 Department of Experimental Psychology, University of Groningen, Groningen, The Netherlands

**Keywords:** Attentional capture, Directed forgetting, Working memory, Cognitive control, Memory-driven capture

## Abstract

Attention is captured by information matching the contents of working memory. Though many factors modulate the amount of capture, there is surprising resistance to cognitive control. Capture occurs even when participants are instructed either that an item would never be a target or to drop that item from memory. Does the persistence of capture under these conditions reflect a rigidity in capture, or can properly motivated participants learn to completely suppress distractors and/or completely drop items from memory? Surprisingly, no studies have looked at the influence of extensive training of involuntary capture from working memory items. Here, we addressed whether training leads to a reduction or even elimination of memory-driven capture. After memorizing a single object, participants were cued to remember or to forget this object. Subsequently, they were asked to execute a search task. To measure capture, we compared search performances in displays that did and did not contain a distractor matching the earlier memorized object. Participants completed multiple experimental sessions over four days. The results showed that attentional capture by to-be-remembered distractors was reduced, but not eliminated in subsequent sessions compared with the first session. Training did not impact capture by to-be-forgotten objects. The results suggest observable, but limited, cognitive control over memory-driven capture.

Living in a world overloaded with information, we need to continually filter out irrelevant aspects of the environment and focus on input that is important for the current goals. Such selection in perception improves the detection, discrimination, and encoding of sensory information (Dosher & Lu, 2000; Jackson et al., 2017; Kastner & Pinsk, 2004; Luck & Hillyard, 1994; Luck et al., 1996; Mangun & Hillyard, 1991; Posner & Petersen, 1990; Turk-Browne et al., 2013). Besides selection in perception, our mind can also prioritize information in memory (Souza & Oberauer, 2016). Selected memories are represented with higher precision and they are more likely to be remembered (Bays & Taylor, 2018; Lin et al., 2021; Makovski et al., 2008; Matsukura et al., 2007; Pertzov et al., 2013; Souza & Oberauer, 2016; Souza et al., 2015, 2016).

These forms of selection dynamically interact with each other (Awh et al., 2012; Awh & Jonides, 2001; Chun & Turk-Browne, 2007; Zanto et al., 2011). Attended items get automatic access to working memory (WM; Hickey et al., 2006; Itti, 2005; Theeuwes, 2010). On the other hand, information voluntarily activated in WM can guide external selection (Gazzaley & Nobre, 2012; Wolfe, 2021; Wolfe & Horowitz, 2004). These findings are consistent with the idea that attention and memory are intertwined processes, supporting each other in a reciprocal fashion (Olivers et al., 2011; Sasin & Fougnie, 2020).

However, the reciprocity between attention and memory can lead to undesired consequences. Specifically, it has been shown that perceptual stimuli matching representations held in WM automatically attract visual attention even when it is disruptive to an immediate task (Downing, 2000; Han & Kim, 2009; Olivers, 2009; Olivers et al., 2011; Pashler & Shiu, 1999; Sasin et al., 2015; Soto et al., 2005; Soto & Humphreys, 2007, 2009). In a typical memory-driven capture paradigm, people are first asked to remember some object (e.g., a green square), which is followed by an unrelated search task. The memory-driven capture is manifested by slower searches when one of the distractor objects in the search display matches WM content (e.g., the object is green) compared with when none of the objects in a display share the feature with an object held in WM. This capture is observed even when the memory-matching distractor is 100% invalid, i.e., it never contains the target (Soto & Humphreys, 2008, 2009). Conversely, the benefits reflected by faster search can be observed when the memory-matching object in the search display contains a target (Balani et al., 2010; Carlisle & Woodman, 2011b).

This bias of attention to items that match the contents of WM can lead to costs or benefits depending on whether the WM representation matches the distractor or the target, respectively. But can cognitive control modulate those costs and benefits? Previous studies seem to provide an affirmative answer to this question by showing that strategic control can diminish the costs or enhance benefits resulting from memory-driven capture (Carlisle & Woodman, 2011b; Dowd et al., 2014; Kiyonaga et al., 2012). For example, Kiyonaga et al. (2012) found that the strength of memory-driven capture is modulated when participants are informed in advance about the predictability of trials in the upcoming block. Specifically, they found that capture by memory-matching invalid distractors was smaller on the block with 100% invalid trials than on the block with 50% invalid and 50% valid trials. This finding suggests that, to some extent, people can control the allocation of attention to WM matching objects. However, critically, the findings also suggest that this control is limited. Even when participants were told that the block would contain only invalid trials, there was still capture.

This persistence of WM capture may be a consequence of a limitation in the architecture of cognition–attention and working memory may draw on equivalent representational and neural substrates. This could reflect the fact that attention and WM share the same top-down control processes (Awh et al., 2006; Chun, 2011; Gazzaley & Nobre, 2012; Postle, 2006; Sahan et al., 2016). In this case, it would be impossible to keep something active in WM without also boosting that information for attention (Downing, 2000). Alternatively, it may be that there does exist some flexibility to completely disengage from capture, but that this can occur only under certain circumstances.

The goal of the present work was to explore whether *training* under conditions in which participants are motivated to avoid capture by relevant WM content could reduce or even eliminate it. This is important because theoretical frameworks connecting attention and WM tend to frame the interaction between attention and WM as arising due to an inflexible overlap in the neural substrates for each task, which is not likely to be modulated by training. Thus, an exploration of whether WM capture is improved by training can help to constrain theoretical frameworks by better understanding the rigidness or flexibility of attentional capture. Moreover, previous studies showed that allowing participants to drop information from memory results in reduced capture if that information is used as search distractors (Sasin & Fougnie, 2020; Sasin et al., 2017). Importantly, the instruction to remove an item from memory may reduce but not eliminate capture (Sasin et al., 2017; but see Olivers et al., 2006). Thus, by applying a training paradigm in the current study, we could also test whether experience and extended practice can improve the ability to eliminate capture from no longer relevant information.

To sum up, so far, studies investigating control over WM capture showed that its magnitude depends on the likelihood of capture being harmful or beneficial for an ongoing task (Carlisle & Woodman, 2011a; Kiyonaga et al., 2012). Moreover, prior work suggests that deprioritization of WM objects in response to forgetting cues significantly reduces WM capture (Sasin et al., 2017). However, no study to date has examined whether extensive training will allow people to exert partial or complete control over capture by distractors matching representations maintained in WM or representations that were activated in WM but are no longer relevant.

In the current study, we used a standard memory capture paradigm (e.g., Sasin et al., 2017), but had participants perform this task over four hour-long sessions on four consecutive days (with no change in instruction) to observe the maximum effects of training. For the task, participants were asked to remember the color and shape of a single item. Next, they were instructed via a retro-cue to *remember* or *forget* this object. A search task was administered during the WM delay that was unrelated to the memory task but during which the memory item could appear at a distractor location (*invalid* trials). On *neutral* trials, the memorized item did not appear in the search display. By tracking the magnitude of attentional capture (i.e., the difference in performance between invalid and neutral trials) across the four sessions, we could test the effect of training on the cognitive control of capture in the *remember* and *forget* conditions. Critically, the task was designed to provide strong motivation to exert cognitive control. First, the memory test executed at the end of the trial was only presented on trials with the remember cue. We did not include invalid memory trials to maximize people’s motivation to forget no-longer relevant objects. Note that including even a small percent of invalid forget cues could result in the intention to keep to-be-forgotten objects in memory (Williams & Woodman, 2012). Second, we did not include valid search trials (i.e., trials on which memory object is presented at a target location) to eliminate the motivation to attend to a matching distractor that could potentially be a target.

To clarify our predictions, we expected that task-irrelevant items matching a memory item would cause capture (slower search when the memory-matching distractor is present in the display than when it is absent). We predicted that capture would be reduced by extensive training. We also predicted that capture would be reduced when participants can forget items from memory. Of particular interest was whether capture could be eliminated, as this would provide evidence of strong top-down control over capture.

## Method

### Participants

Twenty-seven students from the University of Groningen (18 females; nine males; *M* = 20.9 years; *SD* = 2.28) participated in the experiment in exchange for course credit. This sample size was 50% larger than in our previous study (Sasin et al., 2017). We increased the sample size since the requirement to attend four sessions in a row could increase the data’s noise compared with a single session. All participants had normal or corrected to normal visual acuity. The study was approved by the Ethics Committee of the Psychology Department. Informed written consent was obtained.

### Apparatus and stimuli

**Fig. 1:**
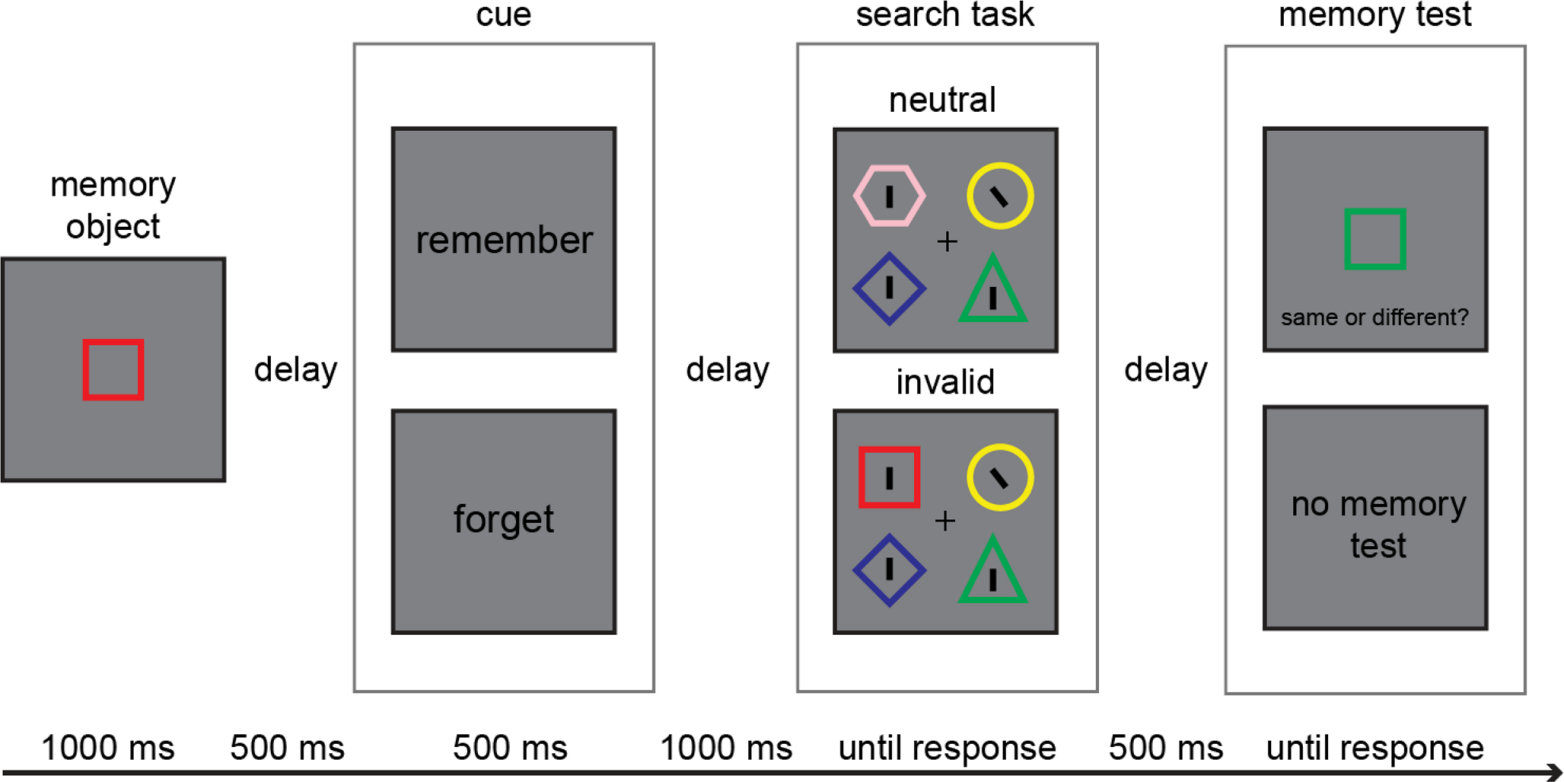
The structure of the task. Presentation of the memory object was followed by the word retro-cue (*forget* or *remember*), which was followed by the search task. On *invalid* trials, one of the search display objects had the same color and shape as the memory object. On *neutral* trials, no object in the search display shared any feature with the memory object. The memory task was present only on trials with the cue *remember*. Participants completed four experimental sessions in four days in a row

Stimuli were presented on LCD monitors (1,920 × 1,080, 60 Hz) with E-Prime 2.0 (Schneider et al., 2002). The shapes used were a circle (1.8° × 1.8° of visual angle), a diamond (1.8° × 1.8°), a square (1.9° × 1.9°), a triangle (2° × 1.7°), and a hexagon (2° × 1.7°). The shapes had slightly different sizes due to the necessity of adding the tilted line target inside the shape. The colors are reported in the CIELAB color space. The color of the shapes could be red (L = 53, A = 80, B = 67), green (L = 88, A = −86, B = 83), blue (L = 32, A = 79, B = −108), yellow (L = 97, A = −22, B = 95) or pink (L = 84, A = 25, B = 3). All stimuli were presented on a gray background (L = 54, A = 0, B = 0). The thickness of the border line of the shapes was 0.12° in visual angle. One colored shape was presented as a memory object at the center of the screen. The cue to forget or remember was displayed in the form of the corresponding word (“remember” or “forget”) being shown in black Courier New, 20-point font, at the center of the screen. In the search task, four different shape outlines in four distinct colors were displayed. Within the outline of each shape was a black line (0.57° length × 0.12°). The target line was tilted 38° either to the left or to the right, whereas the three distractor lines were vertical. The shapes were positioned at the corners of an imaginary rectangle measuring 5.7° of visual angle horizontally and 4.1° vertically centered on the fixation cross. In the memory test, one colored shape was presented as a memory probe at the screen’s center (see Fig. 1 for a schematic representation).

### Procedure

Each trial began with a fixation cross presented for 500 ms. The fixation cross was then replaced by a memory object that remained on the screen for 1000 ms. Participants were instructed to encode both the color and the shape of the memory object in memory. The memory object was followed by a 500-ms blank interval, which in turn was followed by a retro-cue of 500 ms. The retro-cue indicated whether the memory object had to be *remembered* for a later memory test or whether it had to be *forgotten*. Next, there was a 1,000-ms blank interval, after which the search display appeared. Participants were instructed to identify the orientation of the tilted line (only one line was tilted) by pressing the “Z” key when it was tilted to the left and pressing the “M” key when it was tilted to the right. The search screen remained present until participants made a response. Participants were instructed to respond as quickly and accurately as possible. The lines were contained within task-irrelevant shape outlines (see Fig. 1) of distinct color and shape. There were two search display conditions (see Fig. 1). In the *invalid* condition, one of the outlines had the same color and shape as the memory object. This memory-matching object always contained a distractor line and was presented on the opposite side to the target (either at the top or bottom corner of the imaginary rectangle). The distractor was always presented opposite the target to potentially increase the participant’s motivation to suppress the distractor. That is, suppressing or not attending to the side of the distractor would allow participants to be faster in detecting the target.

Conversely, in the *neutral* condition, none of the objects in the search display matched the memory object’s features. On cue remember trials, participants made a response to the memory test after the search test with a blank interval of 500 ms separating the two responses. The memory probe consisted of a single item that either matched the memory item in both shape and color (same trials) or contained a non-matching color or shape (or both) on different trials. Participants had to press the “Z” on the keyboard when the memory probe was the same as the memorized object. If the memory probe was different (in color, shape, or both features) from the memory object, participants had to press the “M” key. Participants were instructed to complete the memory task as accurately as possible, without time pressure. The memory probe remained onscreen until a response was logged.

Participants performed this study over four hour-long sessions in four consecutive days. A short 32 trial practice session proceeded the experimental session on each day. Due to human error, the first session consisted of 480 experimental trials. Three later sessions consisted of 512 experimental trials. In each session, a short break was allowed after every 64 trials. Each session consisted of an equal number of trials per each of four experimental conditions, 2 (cue: *remember* or *forget*) × 2 (distract match: *invalid* or *neutral*).

## Results

One participant failed to meet our *a priori* criteria of above chance performance in memory and search tasks (the participant was correct on only 53.2% of the trials for the memory task). This participant was excluded from all analyses.

### Search results

#### Search accuracy

In each session, participants performed near ceiling in the search task (*M* = 99%, *M* = 97%, *M* = 95%, and *M* = 94% correct, from the first session to the fourth session, respectively). Because search accuracy was near ceiling, we applied arcsine square root transformation (Hogg & Craig, 1995) to the data. A repeated-measures analysis of variance (ANOVA) on transformed accuracy scores showed a main effect of cue, *F*(1, 25) = 6.80, *p* = .015, ${\eta}_p^2$ = .21., with higher accuracy in the cue remember condition than cue forget condition. There was also a main effect of session, *F*(3, 75) = 9.11, *p* = .002, ${\eta}_p^2$ = .27, (Greenhouse–Geisser corrected). The accuracy scores did not differ significantly between types of distractor match, *F*(1, 25) = 1.93, *p* = .177, ${\eta}_p^2$ = .07. There were no significant interactions (all *F*s < .0.70 and all *p*s > .550). Follow-up post hoc comparisons with the Holm-Bonferroni correction showed that accuracy in the search task decreased significantly from the first session to the third session, *t*(25) = 4.03, *p* < .001, *d* = 0.79, and from the second session to fourth session, *t*(25) = 2.57, *p* = .049, *d* = 0.50. These results confirm that all participants could perform the task at a high level, although performance dropped in later sessions.

#### Search response time

**Fig. 2:**
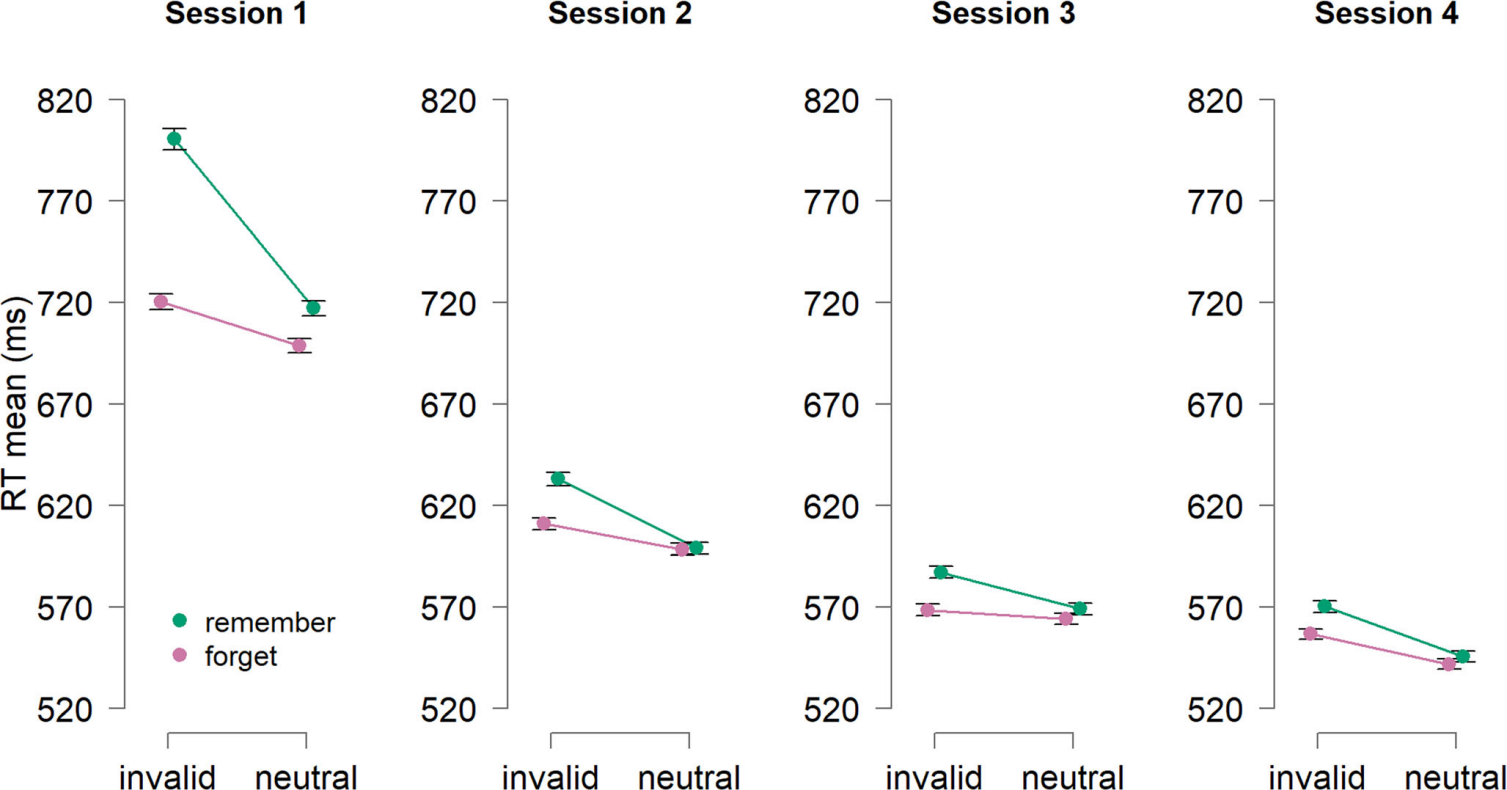
Mean RTs (ms) in the search task as a function of cue condition (*remember* vs. *forget*) and the distractor match (*invalid* vs. *neutral*) across four experimental sessions. Error bars reflect within-subject standard errors of the mean (Morey, 2008)

To examine capture effects, we focus here on the search time as this is the primary measure expected to be affected by capture. Before analyzing the response times (RTs) for the search task, we excluded trials with incorrect memory test responses. Also, we excluded all trials with incorrect responses in the search task. Lastly, we identified and excluded any remaining outliers using the procedure described by Van Selst and Jolicoeur (1994) as in our previous study (Sasin et al., 2017). This resulted in a loss of 2.61% of data points. The exclusion of trials did not change the pattern of results. The results are illustrated in Fig. 2.

#### Capture on remember trials of Session 1

First, we examined whether the search task was affected by the memory matching distractors by comparing the search response times during invalid trials (trials with a memory item at a distractor location) to neutral trials (trials without a memory item) during *remember* trials of the first session. As expected, we find slower search times during invalid trials (800 ms) compared with neutral trials (717 ms), *t*(25) = 6.58, *p* < .001, *d* = 1.29, consistent with previous studies (e.g., Olivers et al., 2006; Soto et al., 2005; Soto & Humphreys, 2009). This demonstrates that the search was impacted by the memory task even though participants knew the distractors were 100% non-predictive of the target.

#### Capture on remember trials with training

**Fig. 3:**
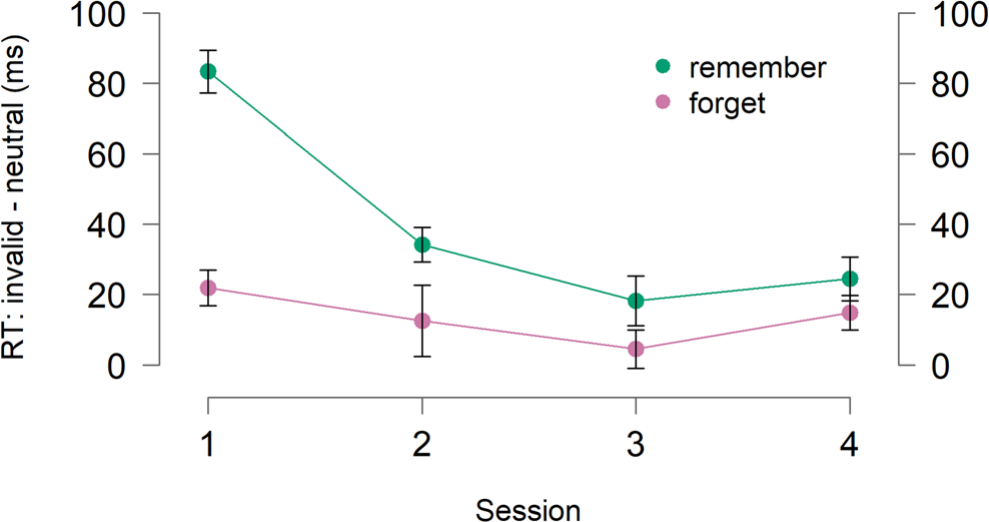
Attentional capture effect (mean invalid RT – mean neutral RT) as a function of cue condition (*remember* vs. *forget*) and the session. Error bars reflect within-subject standard errors of the mean (Morey, 2008)

The central question of this study is to determine whether the capture observed in session 1 remains constant or whether participants got better at avoiding capture with practice. To examine this, we measured the attentional capture effect (mean invalid RTs − mean neutral RTs) separately for each participant and session (Fig. 3) for the remember trials. A repeated-measures ANOVA with the Session factor (1-4) revealed a significant effect of session *F*(3, 75) = 19.14, *p* = < .001, ${\eta}_p^2$ = .43. Follow-up post hoc comparisons with the Holm–Bonferroni correction revealed that the attentional capture score significantly decreased from the first session (83 ms) to the second session (34 ms), *t*(25) = 5.13, *p* < .001, *d* = 1.01.^1^ There were no further changes in attentional capture score across later sessions, all *t*s < 1.69 and all *p*s >.290.

These results demonstrate that memory-driven capture effects are not immutable. With training, participants can learn to better avoid distraction by the memory-matching item. However, after the second session, there was no evidence of an additional benefit of training. Further, the capture did not show much evidence of disappearing completely (there was still significant capture (25 ms) during the final session, *p* < .001). Notably, the effect of training reducing memory-driven capture emerged already in the second session. Thus, one session was sufficient to learn how to improve control over capture. This suggests that people can enhance cognitive control over distraction and do this in a relatively short time. Moreover, the faster search in all conditions suggests that participants can also quickly improve in the search task itself. Possible mechanisms of the training effect are explored in the General Discussion.

#### Capture on forget trials of Session 1

On some trials participants were instructed to drop the stored item from memory (*forget trials*). We found that capture effects were larger on remember (83 ms) relative to *forget* (22ms) trials during Session 1, *t*(25) = 5.39, *p* < .001, *d* = 1.06, suggesting that capture was reduced when the memory item could be forgotten. However, there was still observable capture even on forget trials (720 ms vs. 698 ms, invalid vs. neutral, respectively), *t*(25) = 3.43, *p* = .002, *d* = 0.67.

#### Capture on forget trials with training

Being allowed to drop an item from memory appeared to be beneficial in allowing participants to avoid capture. Does the magnitude of this benefit improve over sessions? To investigate forgetting-based practice (i.e., decrease in capture across sessions because of improved forgetting), we cannot simply examine the magnitude of capture in *forget* trials across sessions, as this comparison ignores the influence of capture-avoidance training effects (i.e., decrease in capture across sessions because improved control over capture). Indeed, the effects of general capture-avoiding practice and forgetting-based practice might be expected to have independent and additive effects (Sternberg, 1969). The prediction of this additive account is that training effects should be *larger* during *forget* trials. If there was no effect of capture-avoiding practice and no effect of forgetting-based practice, the prediction is of a constant benefit of forgetting that does not differ across sessions.

To examine this, we conducted 2 (cue: remember or forget) × 4 (session: 1, 2, 3, 4) repeated-measures ANOVA on capture scores (mean invalid RTs − mean neutral RTs). We found main effect of cue, *F*(1, 25) = 23.45, *p* < .001, ${\eta}_p^2$ = .48 and session, *F*(3, 75) = 16.05, *p* < .001, ${\eta}_p^2$ = .39. The Cue × Session interaction was also significant, *F*(3, 75) = 7.62, *p* < .001, ${\eta}_p^2$ = .23 but the main effect of session was not, *F*(3, 75) = 1.63, *p* = .189, ${\eta}_p^2$ = .06. The interaction suggests that the benefit of forgetting is not additive with training. This finding suggests that training cannot improve control over capture by residual activation from no-longer relevant representations. Moreover, visual inspection of the effect (see Fig. 3) suggests that the effect is *under*-additive, thus indicating that training also did not lead to better forgetting. To test this, we measured the effect of training as the difference in capture effects between sessions, separately for remember and forget trials. We found that the difference in capture effects between the first and last session in forget condition (7 ms) was smaller than the difference in capture effects between the first and last session in remember condition (59 ms), *t*(25) = 3.69, *p* = .001, *d* = 0.72. Similarly, the capture difference between the first and second session was smaller in the forget condition (9 ms) than the remember condition (49 ms), *t*(25) = 3.43, *p* = .002, *d* = 0.67. These findings suggest that there was neither effect of capture-avoiding practice nor forgetting-based practice.

We find no evidence of less capture in later sessions *specifically* in forget trials. Indeed, the benefit of forget compared with remember trials is largest in Session 1 and decreases in subsequent sessions. There are a few possibilities for why this might be the case. One possibility is that participants are improving from training in the *remember* condition by learning to *actively* suppress the memory item, rather than passively ignore it. Logically, the active suppression of an item requires memory of it. Thus, forgetting and active suppression may be two opposing strategies to mitigate capture and would be expected to produce an under-additive effect. A second, nonmutually exclusive possibility is that the under-additive effect occurs due to hitting a floor of capture. Critically, this floor is not zero (there is still significant capture in the last session, *p* = .039). However, the lower the cost of capture means less room to have an impact on overall RT. It is possible that there may be two components to capture—a voluntary component that can be reduced by training in cognitive control and an involuntary component that represents a floor of costs even under optimal cognitive control. It is reasonable to consider that the involuntary component may be linked to priming (Henson, 2003; Kristjánsson & Campana, 2010; Theeuwes, 2013; Tulving & Schacter, 1990). However, it should also be noted that previous work suggests that priming alone does not lead to the capture and that the active maintenance of an item in WM is essential to observe the attentional capture effect (Kumar et al., 2009; Olivers et al., 2006; Soto et al., 2005). Nevertheless, the possibility of priming contribution to the capture cannot be excluded entirely. But even in this possibility, it would still be in support of our argument that there are limitations in the cognitive tools available to avoid attentional capture.

### Memory results

#### Memory accuracy

The performance in the memory task was high, but it seemed to decrease across the sessions (M = 96%, M = 93%, M = 90%, and 89% correct, from the first session to the fourth session, respectively). Again, the arcsine square root transformation was applied to the data. ANOVA on transformed data showed a main effect of session, *F*(3, 75) = 11.24, *p* < .001, ${\eta}_p^2$ = .31. The type of match and interaction between match and session were not significant (all *F*s < 0.98 and all *p*s > .332). Follow-up post hoc comparisons with the Holm–Bonferroni correction showed that accuracy in memory test decreased significantly from the first session to the third session, *t*(25) = 4.29, *p* < .001, *d* = .84., and from the second to the fourth session, *t*(25) = 3.06, *p* = .012, *d* = .60. The memory accuracy changes from the first to the second session, from the second session to the third session, and from the third to the fourth session were not significant (all *t*s < 2.34 and all *p*s > .065). Again, the results confirm that the included participants could perform the memory task at a high level. However, here as well, we observed some drop in accuracy in later sessions.

#### Memory response times

A 2 (match: invalid or neutral) × 4 (session: 1, 2, 3, 4) repeated-measures ANOVA on the mean RTs in the memory task showed the main effect of the session, *F*(3, 75) = 52.00, *p* < .001, ${\eta}_p^2$ = .68. The effect of the match and the interaction between match and session were not significant (all *F*s < 0.55 and all *p*s > .632). Follow-up post hoc comparisons with the Holm–Bonferroni correction showed that RTs in the memory test decreased significantly from the first session to the second session, *t*(25) = 8.13, *p* < .001, *d* = 1.59., and from the second to the third session, *t*(25) = 2.39, *p* = .039, *d* = .47. There was no significant difference between the third and fourth session, *t*(25) = 0.49, *p* = .624, *d* = .10. These findings might suggest that participants prioritize speed more in later sessions. This might explain the drop in memory accuracy. That is, the drop in memory accuracy can result from the speed–accuracy trade-off.

### Speed–accuracy trade-off

The analysis of response times showed that overall, participants responded faster in the second session compared with the first session and in the third session compared with the second session in both search and memory tasks (all *t*s > 2.38 and *p*s < .040). At the same time, we observed a drop in the search and memory accuracy across the sessions. The drop in the accuracy in the memory and search task did not precisely overlap with the change in response times in these tasks. That is, a significant difference in accuracy for both tasks was observed between the first and third sessions and between the second and fourth sessions (all *t*s > 2.56 and *p*s < .05). Nevertheless, we suspect participants prioritize speed over accuracy, and this speed–accuracy trade-off significantly contributed to a drop in accuracy in both tasks. However, there is no evidence that the speed–accuracy trade-off impacted our conclusions about the benefits of training, as these were largely drawn from comparing the first to the second sessions.

## General discussion

In everyday life, we are exposed to an enormous amount of information. However, our attention and memory are severely limited. Fortunately, we can select which information is most relevant through cognitive control. However, there are limits in the effectiveness of this control. Specifically, information activated in WM can guide attention towards matching input even when it disturbs the task at hand. Importantly, in a recent study, we found that cuing people to forget a single object held in WM leads to attenuation of attentional capture by distractors matching to-be-forgotten information (Sasin et al., 2017). This finding showed that no-longer-relevant information can still influence the attentional selection, although this influence is weaker than the influence of relevant information.

Why does capture still persist under circumstances in which the memory item is known to be anti-predictive of the target position and also in cases in which it can be forgotten? Does this reflect the limits of cognitive control, or can capture be further reduced under more optimal conditions? We had participants perform the same memory and search task over four identical hour-long sessions to see if training can reduce capture by distractors matching WM content. Moreover, we examined whether training could eliminate capture by to-be-forgotten objects, so these objects will no longer guide visual selection.

For the data from the first session, we saw capture from the memory item even though participants were instructed that this item would never contain the target. This inability to exert cognitive control replicates a number of studies (Olivers et al., 2011; Pan, 2010; Pashler & Shiu, 1999; Soto et al., 2008). However, rather than reflecting an immutable capture (predicted by some accounts, Soto et al., 2008; Soto & Humphreys, 2007) we see that capture decreased with training. This finding is in line with other studies showing that memory-driven capture can be subjected to strategic control (Dowd et al., 2014, 2015; Kiyonaga et al., 2012; Lu et al., 2017). The improvement from training was only observable from the first to the second session. Further sessions provided no benefits of training, and capture did not completely disappear. This suggests either that some form of capture is inevitable, or at least that the benefits of training are limited.

The current study also looked at the effects of being allowed to drop items from memory and how this would interact with training. We replicated our previous study showing that a retro-cue to forget an earlier encoded item mitigates but does not completely eliminate capture. The effects here can help inform on the benefit of training. One possibility is that participants get better at removing items from memory with increased training, predicting larger differences in capture between memory and forget trials during the later sessions. Alternatively, it could be that the effects of training that we observed in the memory trials were due to participants learning to actively suppress items in memory. Forgetting the memory item may work against the strategy of active suppression and therefore have under-additive effects. Our data are more consistent with the second possibility. Capture differences between the memory and forget trials were largest in the first block and dropped off after that. This was true both in the magnitude of cost produced by capture by to-be-remembered objects as well as proportional forgetting benefits. It is difficult to draw firm conclusions here due to floor effects. However, the data provide no support for the idea that training *improves* cognitive control over capture by to-be-forgotten stimuli. Moreover, we found no evidence that training allows *complete* forgetting of memory items.

Our results help to constrain theories on the mechanisms underlying attentional guidance and control. Broadly, our results are consistent with the idea that storing information in WM involves placing that information in a highly activated state, such that attention will be automatically drawn towards matching information (Larocque et al., 2014; Myers et al., 2017; Olivers et al., 2011). But our findings add to the growing evidence that the resulting capture is not a rigid structural limitation, but something that is sensitive to multiple factors (Houtkamp & Roelfsema, 2006; Olivers, 2009; Olivers et al., 2006; Woodman & Luck, 2007). There are several ways in which training might allow more successful cognitive control. One possibility is that participants learn to suppress items that share features with the stored representation, perhaps by actively inhibiting locations on a guidance map (Noonan et al., 2018; Sawaki & Luck, 2011; Wolfe, 2021), or perhaps by triggering control mechanisms that execute fast disengagement (Lu et al., 2017; Sawaki et al., 2012). Another possibility is that participants are learning to store the memorized information in a state which is less likely to produce capture. For example, state-based models (Oberauer, 2002, 2013) posit that WM consists of both a focus of attention (which exerts a strong influence on visual selection) and latent accessory states that have little influence on visual selection (Larocque et al., 2014; Lewis-Peacock et al., 2012; Myers et al., 2017; Oberauer, 2002, 2013; Olivers et al., 2011). Perhaps a key difference between the first and subsequent sessions is that in later sessions participants are more frequently offloading the WM item to a less active state.

An interesting aspect of our data is that while capture was reduced, there was still observable capture even with extensive training and under ideal conditions to remove capture. This argues that capture is not purely strategic, and that there may be representational and neural overlap between the contents of attention and the information active in WM (Awh et al., 2006; Gazzaley & Nobre, 2012; Sahan et al., 2016). In other words, keeping information active in WM may also place that information as highly active in attentional guidance maps. This is in line with studies showing that once memory-driven capture occurs, the control over this capture can only be applied at later stages of attentional orienting, while the early stage is automatic (Lu et al., 2017; Whitehead et al., 2019). One possibility is that the residual capture we observed in our studies is the initial orienting and the training-based changes in capture reflect differences in the timing and effectiveness of control over capture. Preventing attentional orienting towards memory-matching objects (at least at first) may be challenging to control because, structurally, the mechanisms of attention and WM overlap.

Training participants in a memory-driven capture paradigm revealed that capture can be mitigated but not eliminated. This is consistent with the idea that capture arises because of representational overlap in activated WM and attentional maps. However, there is some cognitive control over this capture, potentially from active suppression of the memory item. Forgetting an item from memory does not completely eliminate capture (even after extensive training), suggesting that stored information is not completely removed from our cognitive system as the result of the retro-cue. Thus, by introducing a training paradigm into a memory-driven capture task we reveal several findings that shape and constrain theory on the relationship between attention and WM. Future work can address the degree to which the training-induced improvement in capture might be general or task-specific learning (e.g., does this benefit transfer to different stimuli or tasks).

The data are available online (https://osf.io/pvur8/).

## Footnotes

1 Note that ANOVA with validity (invalid vs. neutral) and session (1–2) as factors showed that all main effects and the interaction were significant (all *p*s < .001). Post hoc comparisons revealed that RTs decreased from the first to the second session in all conditions (all *p*s < 001).
